# Foreign Summary

**Published:** 1839-11-16

**Authors:** 


### FOREIGN SUMMARY.

*On the Process of Reparation after Simple Fracture of Bones.* By Bransby B. Cooper. (Guy's Hospital Reports, No. vi.)—Twenty-four hours after fracture of the bones, a large quantity of extravasated blood is found effused into the cellular membrane of the muscles, filling up the spaces between the fractured extremities of the bones, and occupying the openings into the cancellated structure of each fractured extremity. The periosteum in the neighborhood of the seat of fracture is also infiltrated with blood and thickened; so that a general extravasation of blood, attended with tumefaction; is all that is to be observed as the immediate result of the injury.

The serous and red colouring matters of the blood now become absorbed, and shortly afterwards inflammatory action commences, which gives rise to the deposition of coagulable lymph. This adheres firmly, not only to the periosteum, but also to the coagulum, which has now acquired a considerable increase in firmness, so as to produce a degree of stiffness of the limb which maintains the bone in a state of comparative rest. The effusion of lymph proceeds so as to fill the adjacent cellular membrane, to occupy the space between the separated fractured extremities of the bone, to thicken the periosteum, to fill up the interstices between the muscular fibres, and, in fact, to present so homogeneous a mass as to render it difficult to distinguish the various structures from each other. About this period the fractured extremities of the bones are found softened, granular, with their asperities partly removed, and firmly adhering to the surrounding lymph.

Blood-vessels now begin to be traced through the surrounding lymph, and an apparent anastomosis is established between the nutrient blood-vessels of the bone, those of the periosteum, and of the cellular membrane surrounding the lymph. A greater degree of firmness is also observed in the direction of the blood-vessels. This altered character of the effusion is most remarkable in the space between the fractured bones, where the lymph puts on the appearance of ligamentous bands more than that of cartilage. The whole mass, however, soon hardens, and forms what is termed *callus.*

Contraction of the callus, apparently the result of interstitial absorption, now commences, and continues till it produces a perfect contact of the overlapping extremities of the bone. A distinct cellular membrane may be observed between the muscles, forming a complete membranous covering to the callus, and continuous with the periosteum of the shaft of each portion of bone to some extent beyond the seat of the fracture.

Between the bones, the callus now puts on the appearance of true cartilage, and at the point of contact no appearance of periosteum can be discovered. This interosseous cartilage is next converted into bone. Several red spots or discs are observed scattered irregularly through it, and round each of these, osseous mattter is deposited, which gradually extends through the whole mass. The extremities of such portions of the shaft as overlap each other, are now found to have lost the compact, and to have assumed a cancellated structure; so that if a longitudinal section be made through the fractured portions, the newly formed bone is found to be continuous with the cancellated structure of each portion of the fractured bone. The whole medium of union is, at this period, enclosed in one continuous investing membrane, but no medullary cavity is yet formed.

The bone now grows less vascular, and a modelling process is established, by which the size of the adventitious deposit becomes reduced. The asperities of the bone are rounded off, grooves are formed for the passage of tendons, blood-vessels, and nerves; and, finally, the medullary cavity is restored, when the process of reparation may be regarded as completed.

*Edin. Med. and Surg. Journ.*

In the Edinburgh Medical and Surgical Journal, for October, there is an interesting article by Dr. W. Thomson, on the Inflammatory Affections of the Internal Organs after External Injuries and Surgical Operations. The following are his conclusions:

"1. That individuals labouring under a chronic affection of any internal viscus, are liable to have an acute inflammatory attack induced in that viscus, by local injuries of remote parts of the body.

2. That inflammatory affections of different organs and textures are liable to occur in individuals who have suffered local injuries, but in whom there is no reason to suppose any disease of these viscera to have existed previously.

3. That different viscera are liable to be affected in different cases of injury of the same part of the body; and that, on the other hand, the same viscera may become affected in cases of local injury of different parts of the body.

4. That in many instances of local injury, pus is effused into remote organs, though suppuration has not occurred in the seat of the primary injury.

5. That the occurrence of affections of remote organs in cases of this nature is generally accompanied by some change in the appearance of the primary injury—as the cessation of the effusion of pus in cases in which suppuration had commenced.

6. That these secondary affections of remote organs occur at very different intervals of time after the reception of the primary injury.

7. That symptoms occasionally occur in cases of this nature, that enable the practitioner to determine which organ is affected—as cough, when the lungs, and jaundice when the liver becomes inflamed.

8. That in most instances, however, the progress of the disease in the remote vicera is very insidious, and affords few or no indications of its existence.

9. That the most probable mode of preventing the occurrence of inflammation in remote organs, subsequently to injury or amputation, is to moderate the constitutional inflammatory tendency, which local injuries produce to a greater or less degree, and particularly to direct these precautions to the organs that may be known to be predisposed to disease, or that show any tendency to become affected."

---

*Substitute for Jalap.*—The seeds of the *Ipomea cœrulea*, the kaladana, or mirahai of the Indian bazars, one of the Convolvulaceæ, which grows abundantly in every hedge and jungle in Bengal, when reduced to powder, and given in doses of from twenty to thirty grains, purges freely, without griping, in from two to three hours. It has been extensively tried in the Native and Police Hospitals of Calcutta, and has been in every case found superior to jalap; inasmuch as it is nearly tasteless, and operates freely, without producing the griping which jalap almost invariably occasions.—*Quarterly Journal of the Calcutta Medical and Physical Society*, July, 1838.

